# Pharmacodynamic interaction analysis of dydrogesterone, progesterone, and estradiol in combination-progestin HRT frozen embryo transfer: a prospective clinical cohort study

**DOI:** 10.1186/s12958-026-01569-2

**Published:** 2026-06-01

**Authors:** Tanja K. Eggersmann, Noemi Hamala, Roman Alexander Friedrich Hiller, Marion Depenbusch, Askan Schultze-Mosgau, Philippos Edimiris, Dunja Baston-Buest, Alexander P. Bielfeld, Jan-Steffen Kruessel, Sören von Otte, Wiebe Junkers, Sascha Tauchert, Reinhard Vonthein, Georg Griesinger

**Affiliations:** 1 University of Luebeck, Campus Luebeck, Ratzeburger Allee 160, 23538 Luebeck, Germany; 2 Universitaeres Kinderwunschzentrum Luebeck and Manhagen, Luebeck, Germany; 3 Department of Reproductive Medicine and Gynecological Endocrinology, University Hospital Schleswig-Holstein, Campus Luebeck, Luebeck, Germany; 4 Department of Obstetrics, Gynecology and REI (UniKiD), Medical Faculty, University Hospital Düsseldorf, Heinrich Heine University, Düsseldorf, Germany; 5 Department of Reproductive Medicine and Gynecological Endocrinology, University Hospital of Schleswig-Holstein, Campus Kiel, Universitaeres Kinderwunschzentrum Kiel, Kiel, Germany; 6 Center for Reproductive Medicine, IVF SAAR Saarbruecken-Kaiserslautern, Saarbrücken, Germany; 7 University of Luebeck, Institute of Medical Biometry and Statistics, Campus Luebeck, Ratzeburger Allee 160, Luebeck, 23538 Germany

**Keywords:** Frozen-thawed embryo transfer cycle, Luteal phase support, Dydrogesterone, Progesterone, Progestin

## Abstract

**Background:**

In anovulatory hormone replacement therapy frozen embryo transfer (HRT-FET) cycles, reproductive success depends entirely on exogenous sex-steroid supplementation. Inadequate progesterone exposure remains a clinically relevant challenge, affecting up to one-third of patients receiving micronized vaginal progesterone (MVP) monotherapy. Combination regimens incorporating oral dydrogesterone (DYD) alongside MVP have been proposed to address this limitation. Critically, the absence of immunoassay cross-reactivity between DYD and progesterone enables simultaneous quantification of both progestins within a single patient – a methodological opportunity not yet exploited in outcome research. Using high-performance liquid chromatography tandem mass spectrometry (HPLC–MS/MS), we investigated the independent and joint associations of DYD, dihydrodydrogesterone (DHD), progesterone (P), and estradiol (E2) with clinical outcomes in a combination-progestin HRT-FET regimen, establishing a novel pharmacodynamic interaction framework for HRT-FET protocol optimization.

**Methods:**

This nested analysis included 111 women undergoing anovulatory HRT-FET within a prospective multicenter cohort (NCT03507673). Patients received oral estradiol (2 mg tid) followed by MVP (400 mg bid) and DYD (10 mg tid) from days 13–15. Serum and plasma samples collected on the day of FET were analyzed using HPLC–MS/MS for DYD and DHD, and immunoassay for P and E2. Clinical pregnancy and live birth were assessed as primary outcomes. Stratified and interaction analyses were performed to explore combined hormone-level effects.

**Results:**

Hormone concentrations showed broad interindividual variability with weak inter-analyte correlations (r ≤ 0.33), confirming pharmacodynamic independence of the two progestin pathways. No statistically significant independent association with live birth was observed for any single analyte. However, interaction analyses revealed consistent gradient patterns: live birth rates were highest when both DYD and P concentrations were elevated (67%) and lowest when both were in the lower range (27%). Analogous patterns were observed for DYD–E2 and P–E2 combinations, suggesting additive and substitutive pharmacodynamic interaction effects. Given the hypothesis-generating nature of this study, the sample size is appropriate for the exploratory interaction framework established here.

**Conclusions:**

This study introduces simultaneous dual-progestin quantification as a methodological platform for pharmacodynamic interaction research in HRT-FET. Exploratory interaction patterns between both progestins and estradiol suggest clinically relevant additive effects, while patterns between the two progestins are compatible with potential substitutive dynamics that can only be evaluated within combination regimens without analytical cross-reactivity. These findings provide a mechanistic framework and generate hypotheses for adequately powered prospective studies investigating joint hormone exposure and reproductive outcomes.

**Trial registration:**

NCT03507673.

**Supplementary Information:**

The online version contains supplementary material available at 10.1186/s12958-026-01569-2.

## Background

Frozen-thawedembryo transfer (FET) cycles account for a substantial proportion of embryo transfers worldwide [35]. FET can be performed in ovulatory or anovulatory regimens. In anovulatory protocols—also referred to as hormone replacement therapy (HRT), programmed, or artificial cycles—follicular development, ovulation, and corpus luteum formation are suppressed or bypassed by exogenous estradiol administration, while endometrial transformation and the implantation window are induced by progestin supplementation. Consequently, implantation and early pregnancy depend entirely on exogenous sex-steroid support in the absence of endogenous luteal progesterone production. If pregnancy occurs, hormonal support must be continued through the early first trimester until placental steroidogenesis is established [25, 33, 34].

Programmed FET cycles remain clinically important, but concerns have been raised regarding both efficacy and safety [27, 41]. First, the absence of a corpus luteum has been associated with adverse maternal and perinatal outcomes in programmed cycles compared with ovulatory approaches [5, 7, 9, 15, 19, 31, 37, 38]. Second, under standard micronized vaginal progesterone (MVP) monotherapy, systemic progesterone exposure varies widely, and low serum progesterone concentrations on or around the day of embryo transfer have repeatedly been associated with reduced reproductive outcomes [8, 20, 21, 28, 29]. Similarly, in a previous prospective HRT-FET cohort using oral dydrogesterone (DYD) 10 mg three times daily as progestin monotherapy, women in the lowest DYD quartile had substantially lower ongoing pregnancy rates than women with higher DYD exposure (−22% absolute difference; 95% CI: −32 to −12, *P*< 0.0001) and a negative interaction of low DYD with low E2 levels was suggested [32].

To improve luteal adequacy in programmed FET cycles, two general strategies have emerged. One is an individualized “screen-and-act” approach, in which in a progestin monotherapy regimen with MVP, serum progesterone is measured during luteal phase around FET and a progestin is added to women with low serum levels [2, 4, 10, 11, 21, 26, 30, 40]. The other is the use of combination regimens in which two progestins are administered simultaneously for endometrial transformation and luteal support. Combination luteal support with oral DYD and MVP has attracted increasing interest as documented in both prospective and retrospective studies [23, 42–45]. Clinically, such regimens may reduce the consequences of inadequate systemic progesterone exposure under vaginal progesterone monotherapy. Methodologically, they offer a unique analytical opportunity because DYD does not cross-react in progesterone immunoassays, allowing simultaneous assessment of two analytically distinct progestin pathways within the same patient [12, 32, 34].

The present study builds on our previous work on DYD monotherapy in anovulatory HRT-FET cycles [32] and now investigates a combination-progestin HRT-FET protocol using oral DYD and MVP. By quantifying DYD, dihydrodydrogesterone (DHD), progesterone (P), and estradiol (E2) on the day of FET, we aimed to explore whether reproductive outcome in HRT-CP-FET is better understood as a function of isolated hormone thresholds or of combined hormone exposure patterns. Given the exploratory nature of this cohort, the objective was not to establish causal interaction effects, but to generate a clinically relevant framework for future studies on multivariable hormone adequacy in programmed FET cycles.

## Materials and methods

### Design and setting

This prospective, observational, multicentre cohort study was conducted at three university-affiliated centres and one private reproductive medicine centre in Germany between February 2021 and August 2023. The study was embedded within an ongoing prospective platform trial (ClinicalTrials.gov identifier: NCT03507673), initiated on 14 April 2018. The platform trial investigates endocrine dynamics of the luteal phase and early pregnancy [13, 32, 34], as well as the vaginal and endometrial microbiome [16, 24], in women undergoing different frozen embryo transfer (FET) regimens. The present analysis represents a predefined observational cohort within the trial infrastructure.

### Study population and FET regimen

This predefined observational cohort included infertile women undergoing frozen embryo transfer after IVF or ICSI in an anovulatory programmed cycle using a combination-progestin HRT-FET regimen. Endometrial preparation was initiated with oral estradiol valerate 2 mg three times daily (Progynova®, Jenapharm GmbH & Co. KG, Jena, Germany). Reassessment was performed after 13–15 days of estradiol administration.

Women were eligible for inclusion if endometrial preparation was considered adequate and if there was no evidence of spontaneous follicular activity or ovulatory escape at reassessment. For the purpose of this study, spontaneous follicular activity or ovulatory escape was defined as the presence of a follicle ≥ 14 mm and/or serum progesterone ≥ 1.0 ng/mL on cycle day 13–15 of endometrial preparation. Women with uterine malformations or relevant endometrial abnormalities, diagnosed either sonographically or by prior hysteroscopy, were excluded.

Once eligibility had been confirmed, luteal transformation was initiated on the same day with oral dydrogesterone 10 mg three times daily (Duphaston®, Abbott Biologicals B.V., Weesp, The Netherlands) and micronized vaginal progesterone suppositories 400 mg twice daily (Cyclogest®, Gedeon Richter Pharma GmbH, Eschborn, Germany). Thus, all women received the same fixed combination-progestin regimen, corresponding to a total daily DYD dose of 30 mg and a total daily MVP dose of 800 mg.

**Fig. 1:**
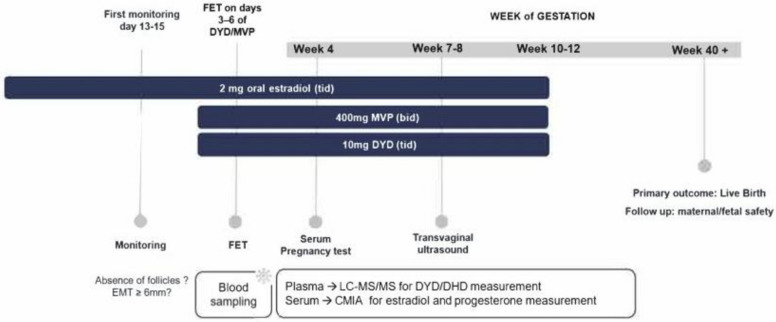
HRT-CP-FET treatment protocol (abbr.: FET, frozen-thawed embryo transfer; EMT, endometrial thickness; HPLC–MS/MS, high-performance liquid chromatography/tandem mass spectrometry; Chemilumineszenz-Mikropartikel-Immunoassay (CMIA) for estradiol and progesterone)

Embryos had previously been vitrified and were warmed using an open manual vitrification system (Kitazato vitrification kit VT601, Gynemed, Lensahn, Germany) [1]. Frozen embryo transfer was scheduled according to embryo developmental stage after 3–6 completed days of combined progestin exposure: on day 3 of DYD/MVP administration for day-2 embryos, day 4 for day-3 embryos, day 5 for day-4 embryos, and day 6 for day-5 embryos. Women who conceived continued estradiol and both progestins into the late first trimester according to local centre policy for anovulatory FET cycles (Fig.1).

### Sampling and outcomes

Blood sampling was performed on the day of frozen embryo transfer, immediately before the transfer procedure. FET was typically scheduled in the late morning. Serum and plasma samples were collected and stored at −80 °C until batch analysis of DYD, DHD, progesterone, and estradiol. A vaginal swab and aspiration of endometrial fluid were also obtained immediately before embryo transfer for separate microbiome analyses.

Sampling on the day of embryo transfer was uniform across centres. However, exact clock time and the interval since the last oral or vaginal hormone administration were not fully standardized and may have contributed to interindividual pharmacokinetic variability. The present study should therefore be interpreted as a pragmatic clinical exposure–outcome analysis rather than as a formal pharmacokinetic profiling study with predefined and, conventionally, serial measurements.

Implantation was defined by a positive serum hCG test 10–14 days after FET according to the local laboratory reference range. Clinical pregnancy was defined as the presence of an intrauterine gestational sac with fetal heartbeat on transvaginal sonography at gestational week 7 or later. After gestational week 7, pregnancy progression, adverse events, live birth, and neonatal outcomes were prospectively followed by structured telephone interviews conducted by a study nurse or doctoral student. Live birth, chosen as the primary outcome for the present analysis, was defined as delivery of a living infant at or beyond the threshold of viability. Maternal and fetal safety of DYD exposed pregnancies is evaluated within the larger cohort across different FET regimens (separate publication in preparation).

### DYD and DHD measurement

The concentration of dydrogesterone and dihydrodydrogesterone (DHD) was determined in 50 μL plasma samples using the High-Performance Liquid Chromatography coupled with Tandem Mass Spectrometry (HPLC/MS/MS) method, which had been previously validated and adjusted to the concentration ranges of 0.050–10.000 ng/mL for DYD and 0.500–100.000 ng/mL for DHD in plasma. Dipotassium ethylenediaminetetraacetic acid (K2EDTA) was used as an anticoagulant. The standard calibration curves covered the ranges of 0.050 to 10.000 ng/mL for DYD and 0.500 to 100.000 ng/mL for DHD. Plasma samples were precipitated with a solution of internal standards dDYD and dDHD (dextrorotatory form of the molecule) in 80% Acetonitrile (ACN). The supernatant was analyzed using by HPLC/MS/MS by company QUINTA-ANALYTICA (Prague, Czech Republic).

### Estradiol and progesterone measurement

Serum estradiol and progesterone were measured using automated immunoassay platforms according to the manufacturers’ instructions. Estradiol was measured using the Alinity i Estradiol assay. Reported intra-assay coefficients of variation ranged from 2.2% to 7.2% across low, medium, and high control levels, and inter-assay coefficients of variation ranged from 2.6% to 7.7%. The limit of detection and limit of quantitation were 20 pg/mL and 24 pg/mL, respectively.

Serum progesterone was measured using the ARCHITECT Progesterone assay. Reported intra-assay coefficients of variation ranged from 1.5% to 5.5%, and inter-assay coefficients of variation were ≤ 6.2% for low controls, ≤ 2.9% for medium controls, and ≤ 3.9% for high controls. The limit of detection was approximately 0.1 ng/mL. The reliable analytical measuring range extended up to 40 ng/mL for progesterone and 1000 pg/mL for estradiol.

Because dydrogesterone does not materially interfere with progesterone immunoassay measurement, this assay strategy allowed simultaneous assessment of serum progesterone and plasma DYD/DHD concentrations within the same cycle.

### Trial objective and sample size

In a previous study, a difference of −22% in the ongoing pregnancy/live birth rate was observed among women undergoing HRT-FET with oral DYD 10 mg (tid) monotherapy, comparing those below and above the 25th percentile of DYD plasma concentrations (odds ratio: 6.09) [32]. The present trial was designed to investigate a putative optimized HRT-CP-FET protocol, with the primary aim of refuting the presence of a similar difference in women with low DYD levels. With a sample size of 111 participants stratified at the 25th percentile, the power to detect the original effect (odds ratio: 6.09) at a one-sided significance level of 5% in an HRT-CP-FET protocol with DYD and additional MVP administration is approximately 80%.

### Statistical analysis

All analyses were predefined as descriptive and exploratory. Continuous variables are presented using mean and standard deviation or by empirical distribution summaries, as appropriate; categorical variables are presented as counts and proportions. For hormone concentrations, empirical distribution functions were examined and, where appropriate, log-transformation was applied prior to further descriptive analysis.

Spearman’s rank-order correlation coefficients were calculated to describe monotonic associations between the measured hormone analytes. To explore clinically interpretable exposure patterns, patients were categorized according to hormone concentrations at embryo transfer using the 25th percentile as a threshold for low versus normal-high exposure. Pregnancy outcomes, including positive pregnancy test, clinical pregnancy, miscarriage, and live birth, were summarized by absolute risk differences with 95% confidence intervals between low and normal-high exposure groups. These descriptive comparisons were stratified by embryo-transfer timing (days 2–3 versus days 4–5) when calculating *P* values.

To explore possible joint hormone exposure patterns, live birth rates were visualized in cross-classified hormone-pair plots using tertiles of hormone concentrations on the day of FET. These visualizations were intended to identify descriptive gradients compatible with additive or partially substitutive exposure–outcome relationships. Given the modest sample size and limited number of live birth events, these analyses were not intended to establish formal interaction effects and should be interpreted as hypothesis-generating. All analyses were performed using jamovi version 2.3.28 [39], Version 2.3.28, Sydney, Australia), R version 4.0.3 (R Core Team. [36], and G*Power 3 version 3.1.9.7 3 (version 3.1.9.7; [14]).

## Results

### Patient flow and demographics

Between February 2021 and August 2023, 728 patients were included across four centres, of whom 111 underwent an embryo transfer in an HRT-CP-FET protocol with plasma and serum samples collected on the day of FET and follow-up to live birth. Supplementary Fig. 1 shows inclusion and patient flow. Supplementary Table 1 summarizes patient demographics. Patients were on average 32.4 years old, weighed 78.3 kg, 96.4% were Caucasian, 38.7% labelled regularly cycling, endometrial thickness of 9.21 mm (± 1.72) and LH levels of 11.6 IU/L (± 8.74) and Progesterone levels of 0.24 ng/ml (± 0.20) on day of last monitoring before prior to initiation of progestin support. FET was performed as single embryo transfer (sFET) in 77.5% of cases and as double embryo transfer (DET) or higher in 22.5% of cases. Clinical pregnancy and live birth rates per woman undergoing FET were 45.0% (95% CI: 36.1% to 54.3%) and 35.1% (95% CI: 26.9% to 44. 4%), respectively.

### Endocrine profile on day of FET 

**Fig. 2:**
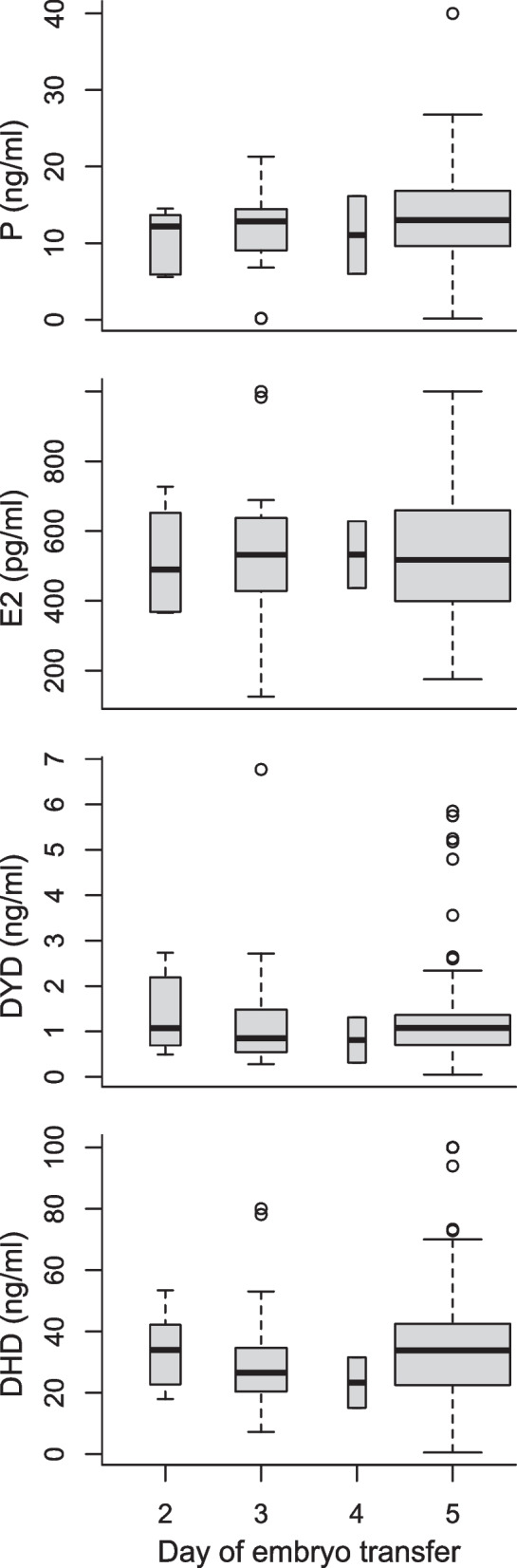
Boxplots of hormone values by day of FET (i.e. by increasing duration of DYD intake and thus cycle progression). For a small number of observations, values were censored at the lower or upper reliable assay limit (DYD *n* = 2, DHD *n* = 6, P *n* = 1, E2 *n* = 4); Abbr.: P = progesterone; E2 = estradiol; DYD = dydrogesterone; DHD = 20α-dihydrodydrogesterone)

Empirical distributions of DYD, DHD, P, and E2 with lognormal or normal distribution functions overlayed are Supplementary Fig. 2. Hormone analyte levels were not associated with cycle progression (Fig. 2). Of note, 22/111 (19.8%) of patients were below a progesterone level of 8.90 ng/ml on day of FET previously reported as a predictive threshold for ongoing pregnancy or live birth in an HRT-FET protocol with MVP [29]. DYD and DHD concentration distributions on the 3rd to 6th day of intake were consistent with previous findings [32] (Supplementary Table 2). Progesterone showed virtually no correlation with E2 (r_P,E_ = −0.03), while DYD and E2 were also essentially uncorrelated (r_DYD,E2_ = 0.09). Correlations of P with DYD and DHD were weak (r_DYD,P_ = 0.23; r_DHD,P_ = 0.33).

### Treatment outcome by hormonal levels on day of FET

**Fig. 3:**
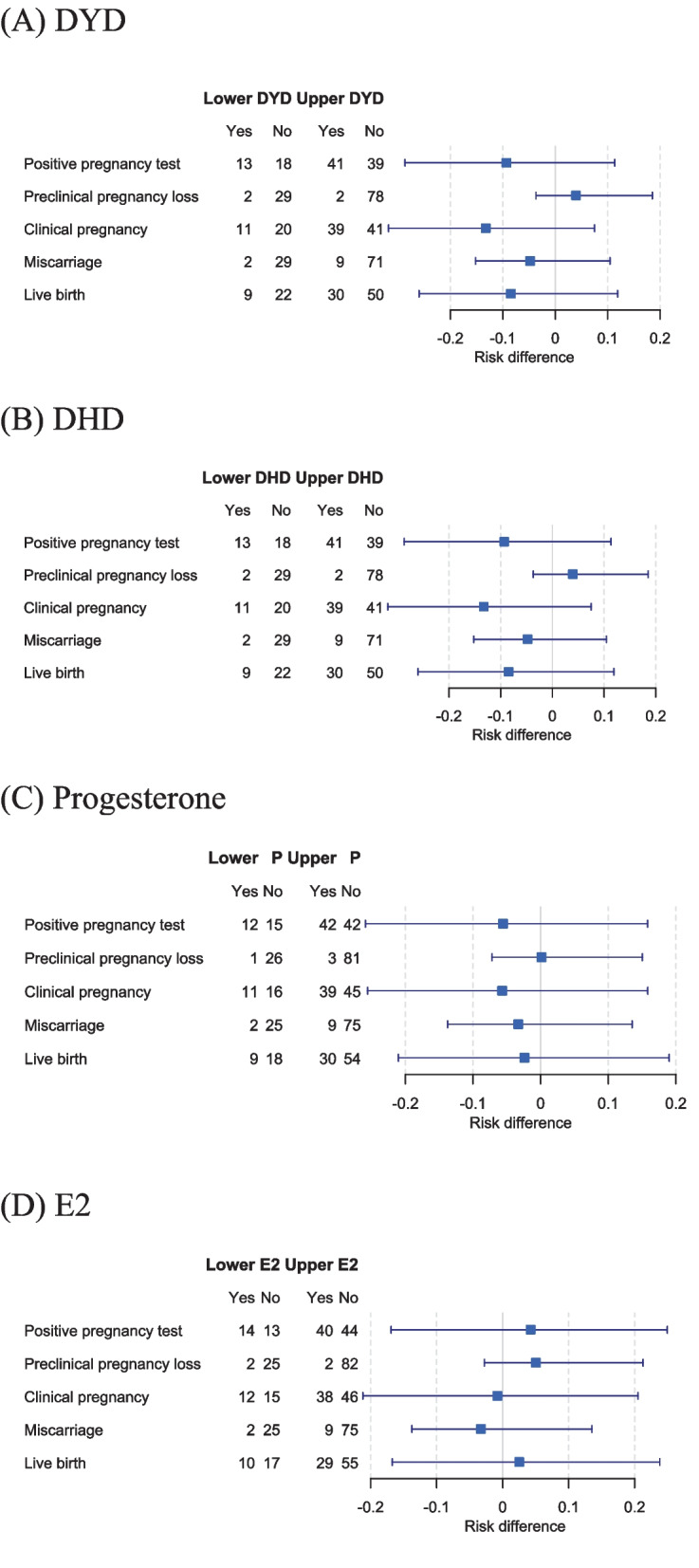
Forest plots with risk differences with confidence intervals for treatment outcome from implantation to live birth calculated per patient undergoing FET, in subgroups of low (≤ 25th percentile) versus normal-high (> 25th percentile) hormone levels on day of FET. (abbr.: **A**, DYD = dydrogesterone; **B**, DHD = 20α-dihydrodydrogesterone; **C**, Progesterone = progesterone; **D**, E2 = estradiol)

Among the 111 patients, 23 underwent embryo transfer on day 2/3 and 88 on day 4/5. The live birth rate was 13.0% (3/23) following day 2/3 transfers and 40.9% (36/88) following day 4/5 transfers (odds ratio 4.56, 95%-confidence interval 1.22 to 25.7, *P* value 0.014). Figure 3 illustrates non-significant risk differences with confidence intervals for implantation, clinical pregnancy, early and late pregnancy loss, and live birth in FET patients, categorized into subgroups with low vs. normal-high hormone levels by quartiles.

**Fig. 4:**
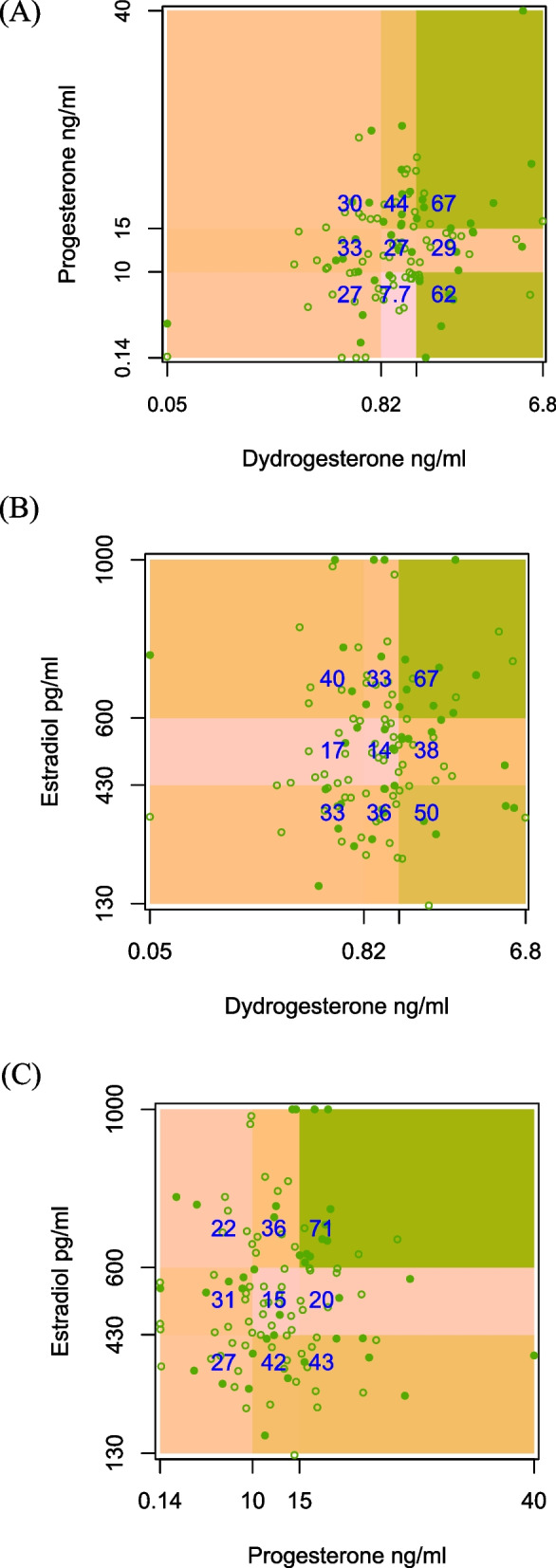
Live birth rates stratified by hormone concentration tertiles on the day of FET. Scatterplots depict combinations (**A**-**C**) of dydrogesterone (DYD), progesterone, and estradiol (E2) levels measured on the day of frozen embryo transfer (FET). DYD concentrations were log-transformed. Each panel shows the observed live birth rates (blue numbers, in %) within the nine resulting strata. Filled green dots represent individual patients achieving live birth

Figure 4 displays scatter plots stratified by (log-)terciles of hormone concentrations measured on the day of FET, overlaid with color-coded live birth rates for each hormone pair. The DYD–P plot indicates an additive pattern: live birth probability is lowest when both DYD and P are low (27%) and highest when both are high (67%). Notably, live birth rates are relatively high (62%) even when P is low, but DYD was elevated, suggesting that DYD may partially substitute for low progesterone exposure. A similar additive pattern was observed for DYD–E2 and a suggestion of synergy between P and E2. If higher levels of sex-steroids were associated with improved live birth probability, DYD might be a decisive contributor when hormonal exposures are discordant.

## Discussion

In this prospective observational study of anovulatory HRT-FET cycles supported by a combination of oral dydrogesterone (DYD) and micronized vaginal progesterone (MVP), plasma DYD levels on the day of embryo transfer below 0.71 ng/ml were not much associated with pregnancy or live birth outcomes. Notably, DYD (and its active metabolite DHD) concentrations showed a distribution comparable to a previous 10 mg DYD tid mono-therapy HRT-FET study [32], in which DYD below 0.71 ng/ml was linked to reduced live birth rates. In contrast, our findings suggest that when DYD is combined with MVP, the predictive value of individual serum hormone levels—whether DYD, progesterone, or estradiol—appears limited. This may imply that routine measurement of only one of these hormones at the time of FET may be unnecessary for guiding clinical decision-making in an HRT-CP-FET protocol.

These data thus corroborate previous observations showing that in HRT-CP-FET cycles with DYD and MVP, serum progesterone levels on the day of embryo transfer are not predictive of live birth. Specifically, [23]retrospectively analysed 560 HRT-FET cycles with euploid embryo transfer in which luteal support consisted of 30 mg/day oral DYD and 300 mg/day MVP. Serum progesterone but not DYD/DHD was measured on the day of FET and in patients with levels < 10 ng/ml MVP dosing was initially increased.However, multivariable logistic regression adjusted for age, BMI, and embryo quality, as well as weighted analyses using inverse probability treatment weights, showed no significant association between serum progesterone and ongoing pregnancy when patients already receive DYD 10 mg tid. Our findings not only support this conclusion but also suggest the mechanism of substitution: when progesterone levels are low, DYD levels appear to compensate, with DYD emerging as a potentially decisive contributor when hormonal exposures are discordant. The latter, however, needs confirmation in larger studies.

A noteworthy observation in our dataset is the presence of several patients with extremely low serum progesterone levels despite administration of 2 × 400 mg MVP. As shown in Fig. 4, five women had values at the lower detection limit of the assay (0.14 ng/ml), suggesting a potential subgroup with markedly impaired vaginal absorption or increased systemic clearance. This finding is concerning, particularly given that MVP monotherapy remains widely used in HRT-FET protocols [18]. While non-compliance cannot be entirely excluded, it appears unlikely in this setting due to the typically high intrinsic motivation of women undergoing fertility treatment. Furthermore, comparable observations have also been reported in other studies using Cyclogest® at 2 × 400 mg/day. Based on the reported means and standard deviations—and assuming a normal distribution of serum progesterone—the estimated proportion of women with levels below 1 ng/mL can be estimated as 0.41% in Labarta et al. [22]*n*= 663, mean = 14.5 ng/mL, SD = 5.1), 1.46% in Alsbjerg, et al. [2]*n*= 488, mean = 15.4 ng/mL, SD = 6.6), 0.26% in Herencia et al. [17]*n*= 131, mean = 13.6 ng/mL, SD = 4.5), and 1.8% in Baldini et al. [6]*n*= 281, mean = 14.0 ng/mL, SD = 6.2). Similar results can be estimated from other large observational studies using vaginal progesterone formulations such as Utrogestan®, Crinone®, and Endometrin® (summarized in [3] Altogether, these findings highlight substantial interindividual variability in systemic progesterone exposure under MVP monotherapy and underscore the potential value of combination progestin strategies in HRT-FET protocols, allowing for substitution between different routes of administration.

In our previous prospective study investigating DYD monotherapy in anovulatory HRT-FET cycles, we already observed a kind of interaction between serum E2 and DYD levels on the day of FET: elevated E2 levels appeared to mitigate the adverse association of low DYD with live birth rate, suggesting a substitution effect between these two hormones (depicted in Fig. 5 in [32]. In the present study, with the addition of a second progestin (MVP) to the luteal support regimen, we now hypothesize a synergistic pattern between Progestins and E2. Live birth rates were consistently highest when both hormones of a given pair (DYD-E2, P-E2) were elevated and lowest when both were low. This observation, if further corroborated, has important ramifications for clinical practice and protocol optimization. Several studies have proposed the use of a single progesterone threshold on the day of FET as predictive of clinical pregnancy or live birth—for instance, [20]suggested a cut-off of 9.2 ng/ml, while other thresholds ranging from 8 to 11 ng/ml have been reported in the literature (see [29] for summary). However, such an approach may be overly simplistic for identifying patients with suboptimal sex-steroid support. Omitting potential interactions between estrogenic and progestogenic sex steroids assumes that each hormone exerts its effect in isolation, which may not reflect the complex endocrine milieu required for optimal endometrial receptivity and pregnancy maintenance. Single threshold-based assessments may indeed also distort smooth effects, misrepresent additive and substitution effects of different hormones and lead to over- and undertreatment in individual cases. This underscores the need for multivariable and interaction-sensitive models in evaluating hormone adequacy in HRT-FET protocols, particularly because all administered sex steroids in this setting are pharmacologic substitutes for endogenous production and display substantial inter-individual variability.

Importantly, conventional logistic regression models—which rely on the logit link function—tend to model multiplicative effects on the odds scale and may obscure additive or substitutive relationships between covariates. When hormonal variables interact in a compensatory manner, such patterns may not be readily apparent in regression coefficients alone. Visual inspection of interaction plots, such as the hormone interaction matrices shown in Fig. 4, can therefore provide valuable complementary insights into complex biological relationships and guide a more nuanced understanding.

A novel and unexpected observation in this study is a potentially additive effect of a DYD and MVP combination, indicating that a higher exposure to both agents may confer a cumulative benefit. While our study was initially designed to explore how one progestin may partially compensate for low levels of the other—implying a substitution effect—an obvious opportunity arises for improving outcomes by optimizing overall progestin exposure, whether through increased dosing, alternative routes of administration, or individualized adjustments. This has obvious and potentially important implications for routine HRT-FET regimens, which have been suspected to carry a higher risk of pregnancy loss after implantation compared to natural cycles. Inadequate progestins exposure may be a contributing factor. Of note, such a phenomenon may not become apparent in pragmatic RCTs measuring mean effects at the population level [18] as this phenomenon may affect only a (too small) fragment of all women treated. Still, by leveraging the potential synergistic effect of dual-progestin strategies, protocols could be refined to ensure more consistent and potentially optimized exposure across patients.

This study has several important limitations. First, the sample size was modest and the number of live birth events limited, restricting statistical power for detecting small effect sizes and precluding robust multivariable or formal interaction modelling. This was partly due to the declining clinical use of HRT-FET protocols, which are no longer considered first-line in many centres for patients eligible for ovulatory FET regimens [41], thereby limiting prospective recruitment. Although approximately 200 HRT-FET cycles had initially been anticipated within the broader study framework, only 111 observations became available. Still, the weak pairwise correlations between DYD, progesterone, and estradiol reduced concerns of multicollinearity and allowed exploratory cross-classification of hormone pairs without substantial redundancy, facilitating the interpretation of possible additive or partially substitutive exposure–outcome patterns. These observations, however, remain hypothesis-generating and cannot replace adequately powered inferential analyses. Second, although sampling on the day of embryo transfer was uniform, exact clock time and time since last dose were not fully standardized, which may have contributed to pharmacokinetic variability. Third, residual confounding cannot be excluded. Centre effects, embryo developmental stage, number of embryos transferred, and embryo-related quality characteristics may all have influenced outcomes; in particular, embryo quality and euploidy status were not uniformly available across all participating centres. Fourth, this cohort did not include complementary endometrial receptivity markers such as endometrial volume, blood flow, cortisol, or prolactin. Accordingly, the present study should be understood as a pragmatic prospective exposure–outcome cohort that generates hypotheses for future validation rather than as a definitive mechanistic analysis.

## Conclusion

In summary, in DYD + MVP-supported HRT-FET cycles, serum progesterone levels on the day of embryo transfer did not appear to predict reproductive outcomes reliably, challenging mono-progestin “screen-and-act” concepts based solely on single progesterone thresholds. The observed interaction patterns—particularly between estradiol and progestins—suggest that single-hormone cut-off models may oversimplify the endocrine interplay underlying endometrial receptivity and early pregnancy support.

By enabling simultaneous and analytically distinct quantification of two progestin pathways, this study provides a useful methodological platform for pharmacodynamic interaction research in HRT-FET. The descriptive patterns observed in this cohort are compatible with additive effects between progestins and estradiol, as well as partially substitutive dynamics between dydrogesterone and progesterone, but these interpretations remain hypothesis-generating.

Future adequately powered prospective studies should therefore evaluate joint hormone exposure rather than isolated single-hormone thresholds and should ideally include larger sample sizes, predefined blood sampling timepoints in relation to the last hormone intake, and more homogeneous treatment settings. In particular, future research should compare different combination-progestin regimens, such as DYD + MVP and DYD + subcutaneous progesterone, and, where feasible, focus on more uniform embryo-transfer conditions, for example single blastocyst transfer only. Such study designs may help to define whether multivariable hormone-based models can improve individualized luteal support in artificial FET protocols.

## Supplementary Information

Supplementary Material 1. Figure S1. A total of 728 women were enrolled across the four participating centres. Of these, 58 were excluded from the analysis for the following reasons: missing blood sample on the day of FET (n=18), withdrawal of consent (n=12), inadequate embryonic development (n=17), and no embryo transfer due to illness (n=11). Of the remaining 670 women who underwent embryo transfer and had evaluable follow-up, 559 underwent programmed-ovulatory FET (PO-FET) and 111 underwent HRT combination-progestin FET (HRT-CP-FET), the latter constituting the analysis cohort of the present study. Figure S2: Empirical and theoretical hormone level distributions on day of FET. Normal distributions were assumed for P and E2, lognormal distributions for DYD and DHD. Vertical reference lines are lower quartiles at embryo transfer. Table S1. Demographics of the analyzed patient population with stratification for live birth achievement; depicted are mean and standard deviation or number and proportions (abbr.: AMH = Anti-Muellerian Hormone; EMT = endometrial thickness; LH = Luteinizing hormone; COCs = cumulus-oocyte-complexes; SET = single embryo transfer; DET = double embryo transfer; *missing values n=1). Table S2. Observed and inferred quantiles of hormone concentrations at different days of frozen embryo transfer (FET), assuming normal distributions of progesterone, estradiol, and of the logarithms of dydrogesterone and 20α-dihydrodydrogesterone (abbr.: DYD = dydrogesterone; DHD = 20α-dihydrodydrogesterone; E2 = estradiol; FET = frozen embryo transfer). Table S3. confirms no statistically significant associations of low vs. normal-high hormone levels by quartiles with these outcomes when stratifying for early (days 2–3) vs. later (days 4–5) FET

## Data Availability

The analysis dataset and statistical code underlying this study are currently being curated and will be made publicly available on Zenodo and GitHub under the dataset title “HRT_CP_FET _Luebeck.” A DOI-linked Zenodo repository will be established to ensure long-term accessibility and transparency. In the meantime, the data are available from the corresponding author upon reasonable request.

## Abbreviations

CAN: Acetonitrile
AI: Artificial Intelligence
AMH: Anti-Müllerian Hormone
bid: Bis in die (twice daily)
BMI: Body Mass Index
CI: Confidence Interval
CMIA: Chemilumineszenz-Mikropartikel-Immunoassay
COCs: Cumulus–Oocyte Complexes
CP: Combination Progestin
CRF: Case Report Form
CV: Coefficient of Variation
DET: Double Embryo Transfer
DHD: 20α-Dihydrodydrogesterone
DOI: Digital Object Identifier
DYD: Dydrogesterone
E2: Estradiol
EMT: Endometrial Thickness
ET: Embryo Transfer
FET: Frozen-Thawed Embryo Transfer
GnRH: Gonadotropin-Releasing Hormone
hCG: Human Chorionic Gonadotropin
HPLC–MS/MS: High-Performance Liquid Chromatography Tandem Mass Spectrometry
HRT: Hormone Replacement Therapy
HRT-CP-FET: Hormone Replacement Therapy Combination-Progestin Frozen Embryo Transfer
ICSI: Intracytoplasmic Sperm Injection
IFFS: International Federation of Fertility Societies
IVF: In Vitro Fertilization
IU: International Units
K2EDTA: Dipotassium Ethylenediaminetetraacetic Acid
LH: Luteinizing Hormone
LoD: Limit of Detection
LoQ: Limit of Quantitation
MVP: Micronized Vaginal Progesterone
ng/mL: Nanograms per Milliliter
P: Progesterone
pg/mL: Picograms per Milliliter
PRISMA: Preferred Reporting Items for Systematic Reviews and Meta-Analyses
RCT: Randomized Controlled Trial
RD: Risk Difference
REI: Reproductive Endocrinology and Infertility
rho (r): Spearman’s Rank Correlation Coefficient
sFET: Single Frozen Embryo Transfer
SET: Single Embryo Transfer
SD: Standard Deviation
tid: Ter in die (three times daily)
VBL: Versorgungsanstalt des Bundes und der Länder
WHO: World Health Organization
