# Supplementary material for: Pharmacodynamic interaction analysis of dydrogesterone, progesterone, and estradiol in combination-progestin HRT frozen embryo transfer: a prospective clinical cohort study
Source: Reprod Biol Endocrinol. 2026 Jun 1;24:57. doi: 10.1186/s12958-026-01569-2 (PMC13227871; doi:10.1186/s12958-026-01569-2)
Supplement: Supplementary file 1 — Supplementary Material 1. Figure S1. A total of 728 women were enrolled across the four participating centres. Of these, 58 were excluded from the analysis for the following reasons: missing blood sample on the day of FET (n=18), withdrawal of consent (n=12), inadequate embryonic development (n=17), and no embryo transfer due to illness (n=11). Of the remaining 670 women who underwent embryo transfer and had evaluable follow-up, 559 underwent programmed-ovulatory FET (PO-FET) and 111 underwent HRT combination-progestin FET (HRT-CP-FET), the latter constituting the analysis cohort of the present study. Figure S2: Empirical and theoretical hormone level distributions on day of FET. Normal distributions were assumed for P and E2, lognormal distributions for DYD and DHD. Vertical reference lines are lower quartiles at embryo transfer. Table S1. Demographics of the analyzed patient population with stratification for live birth achievement; depicted are mean and standard deviation or number and proportions (abbr.: AMH = Anti-Muellerian Hormone; EMT = endometrial thickness; LH = Luteinizing hormone; COCs = cumulus-oocyte-complexes; SET = single embryo transfer; DET = double embryo transfer; *missing values n=1). Table S2. Observed and inferred quantiles of hormone concentrations at different days of frozen embryo transfer (FET), assuming normal distributions of progesterone, estradiol, and of the logarithms of dydrogesterone and 20α-dihydrodydrogesterone (abbr.: DYD = dydrogesterone; DHD = 20α-dihydrodydrogesterone; E2 = estradiol; FET = frozen embryo transfer). Table S3. confirms no statistically significant associations of low vs. normal-high hormone levels by quartiles with these outcomes when stratifying for early (days 2–3) vs. later (days 4–5) FET [file 12958_2026_1569_MOESM1_ESM.docx]

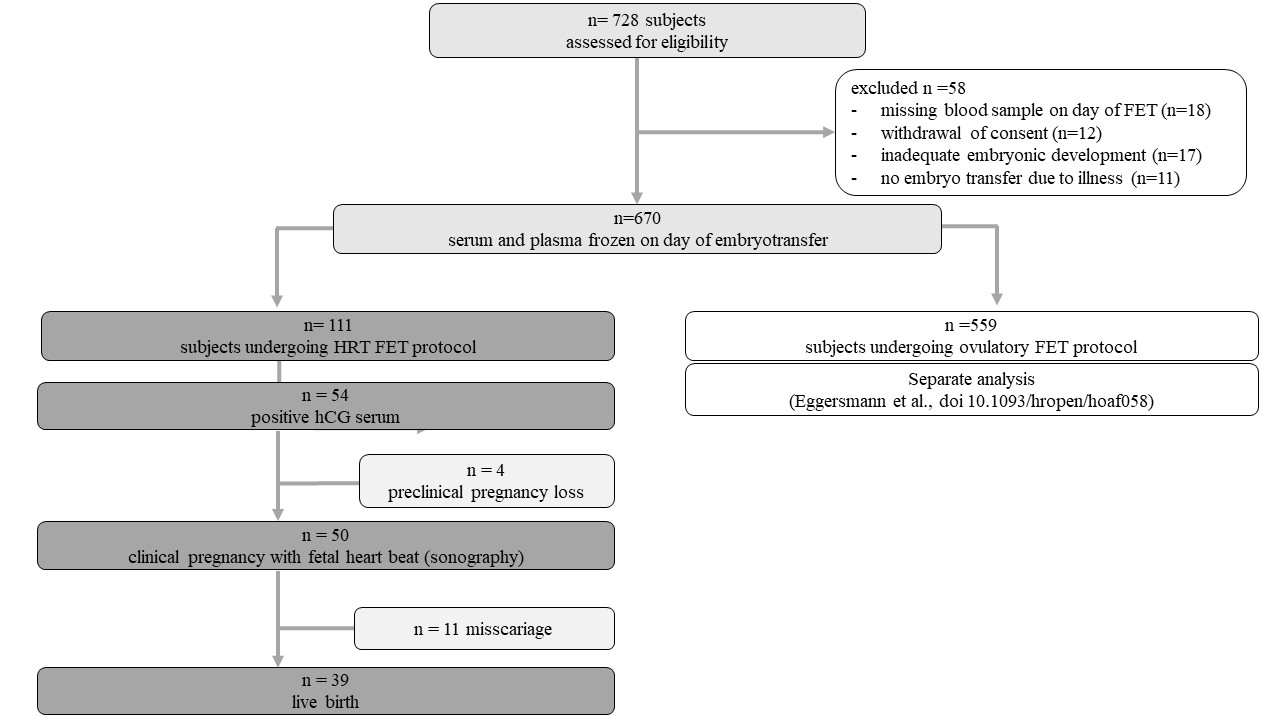


***Figure S1:*** A total of 728 women were enrolled across the four participating centres. Of these, 58 were excluded from the analysis for the following reasons: missing blood sample on the day of FET (n=18), withdrawal of consent (n=12), inadequate embryonic development (n=17), and no embryo transfer due to illness (n=11). Of the remaining 670 women who underwent embryo transfer and had evaluable follow-up, 559 underwent programmed-ovulatory FET (PO-FET) and 111 underwent HRT combination-progestin FET (HRT-CP-FET), the latter constituting the analysis cohort of the present study**.**


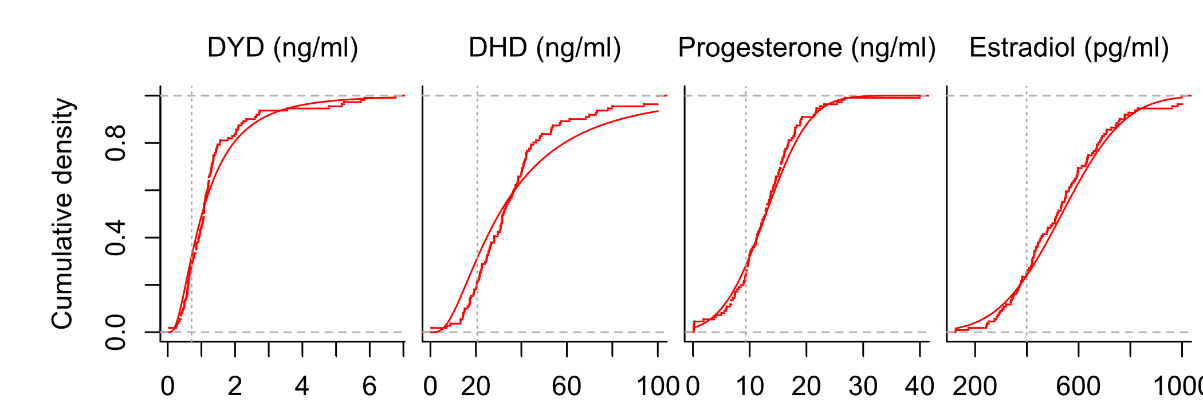


***Figure S2:*** Empirical and theoretical hormone level distributions on day of FET. Normal distributions were assumed for P and E2, lognormal distributions for DYD and DHD. Vertical reference lines are lower quartiles at embryo transfer.

***Table S1:*** Demographics of the analyzed patient population with stratification for live birth achievement; depicted are mean and standard deviation or number and proportions (abbr.: AMH = Anti-Muellerian Hormone; EMT = endometrial thickness; LH = Luteinizing hormone; COCs = cumulus-oocyte-complexes; SET = single embryo transfer; DET = double embryo transfer; *missing values n=1).

|  | **Overall (n=111)** | **No live birth (n=72)** | **Live birth (n=39)** |
| --- | --- | --- | --- |
| **Age at follicular puncture (years)** | 32.4 (3.56) | 32.8 (3.69) | 31.7 (3.22) |
| **Height (cm)** | 168 (6.71) | 168 (6.33) | 168 [7.45] |
| **Weight (kg)** | 78.3 (19.4) | 79.0 (19.7) | 77.1 (18.9) |
| **Body mass index (kg/m²)** | 27.6 (6.37) | 27.9 (6.36) | 27.1 (6.44) |
| **Duration of infertility (months)** | 48.0 (33.4) | 52.3 (35.1) | 40.2 (28.8) |
| **Caucasian, n (%)** | 107 (96.4) | 68 (94.4) | 39 (100.0) |
| **Eumenorrheic, n (%)** | 43 (38.7) | 35 (48.6) | 8 (20.5) |
| **AMH (ng/ml)** | 5.19 (3.68) | 5.05 (3.81) | 5.45 (3.46) |
| **Smoking, n (%)** | 23 (20.7) | 18 (25.0) | 5 (12.8) |
| **Polycystic ovarian syndrome, n (%)** | 33 (29.7) | 20 (27.8) | 13 (33.3) |
| **Endometriosis, n (%)** | 16 (14.4) | 10 (13.9) | 6 (15.4) |
| **COCs (n)** | 17.1 (7.68) | 16.5 (7.98) | 18.3 (7.03) |
| **Trigger Medication***  **hCG, n (%)**  **GnRH-Agonist, n (%)** | 72 (69.5)  38 (30.6) | 50 (69.4)  22 (30.6) | 22 (57.9)  16 (42.1) |
| **EMT at last monitoring (mm)** | **9.21 (1.72)** | **9.08 (1.48)** | **9.45 (2.11)** |
| **LH at last monitoring (IU/l)** | 11.6 (8.74) | 11.3 (9.92) | 12.3 (6.28) |
| **Progesterone at last monitoring (ng/ml)** | 0.24 (0.20) | 0.26 (0.23) | 0.204 (0.14) |
| **SET, n (%)** | 86 (77.5) | 55 (76.4) | 31 (79.5) |
| **DET or higher, n (%)** | 25 (22.5) | 17 (23.6) | 8 (20.5) |

***Table S2:*** Observed and inferred quantiles of hormone concentrations at different days of frozen embryo transfer (FET), assuming normal distributions of progesterone, estradiol, and of the logarithms of dydrogesterone and 20α-dihydrodydrogesterone (abbr.: DYD = dydrogesterone; DHD = 20α-dihydrodydrogesterone; E2 = estradiol; FET = frozen embryo transfer).

| **Observed quantiles of hormone concentrations on different days of FET** | | | | | | | | | | | | | | | | | | | | | | | | | | | | | | | | |  |  |  |  |  |  |  |  |
| --- | --- | --- | --- | --- | --- | --- | --- | --- | --- | --- | --- | --- | --- | --- | --- | --- | --- | --- | --- | --- | --- | --- | --- | --- | --- | --- | --- | --- | --- | --- | --- | --- | --- | --- | --- | --- | --- | --- | --- | --- |
| **Day of FET** | **DYD (ng/ml)** | | | | | | | **DHD (ng/ml)** | | | | | | | | | **Progesterone (ng/ml)** | | | | | | | | **E2 (pg/ml)** | | | | | | | |  |  |  |  |  |  |  |  |
|  | **5%** | **25%** | | **50%** | **75%** | | **95%** | **5%** | | **25%** | | **50%** | **75%** | | **95%** | | **5%** | **25%** | | **50%** | | **75%** | **95%** | | **5%** | | **25%** | **50%** | | **75%** | | **95%** |  |  |  |  |  |  |  |  |
| **2** | 0.5420 | 0.779 | | 1.070 | 1.921 | | 2.598 | 19.16 | | 25.23 | | 33.95 | 40.41 | | 50.61 | | 5.648 | 7.43 | | 12.18 | | 13.33 | 14.30 | | 366.2 | | 376.1 | 489.8 | | 634.6 | | 708.6 |  |  |  |  |  |  |  |  |
| **3** | 0.3750 | 0.545 | | 0.847 | 1.484 | | 3.526 | 12.04 | | 20.42 | | 26.51 | 34.63 | | 78.50 | | 0.174 | 9.03 | | 12.83 | | 14.46 | 16.92 | | 273.7 | | 428.0 | 531.8 | | 637.2 | | 986.6 |  |  |  |  |  |  |  |  |
| **4** | 0.3646 | 0.563 | | 0.811 | 1.059 | | 1.257 | 15.91 | | 19.21 | | 23.33 | 27.45 | | 30.75 | | 6.478 | 8.51 | | 11.05 | | 13.59 | 15.62 | | 445.7 | | 484.1 | 532.0 | | 579.9 | | 618.3 |  |  |  |  |  |  |  |  |
| **5** | 0.4078 | 0.705 | | 1.076 | 1.360 | | 4.485 | 14.26 | | 22.55 | | 33.86 | 42.41 | | 88.80 | | 3.843 | 9.60 | | 13.00 | | 16.81 | 22.98 | | 277.6 | | 400.0 | 517.4 | | 653.0 | | 826.6 |  |  |  |  |  |  |  |  |
| **Inferred quantiles of hormone concentrations on different days of FET assuming normal distributions** **of P, E2 and of logarithms of DYD and DHD.** | | | | | | | | | | | | | | | | | | | | | | | | | | | | | | | | | | | | | | | | |
| **Day of FET** | | | **DYD (ng/ml)** | | | | | | | | | | | | | **DHD (ng/ml)** | | | | | | | | | | | | | **Progesterone (ng/ml)** | | | | | | | **E2 (pg/ml)** | | | | |
|  |  |  | **5%** | | | **25%** | | | **50%** | | **75%** | | | **95%** | | **5%** | | | **25%** | | **50%** | | | **75%** | | **95%** | | | **5%** | | **25%** | | **50%** | **75%** | **95%** | **5%** | **25%** | **50%** | **75%** | **95%** |
| **2** | | | 0.4296 | | | 0.7688 | | | 1.152 | | 1.726 | | | 3.089 | | 17.52 | | | 24.97 | | 31.93 | | | 40.83 | | 58.17 | | | 4.762 | | 8.244 | | 10.66 | 13.09 | 16.57 | 277.4 | 417.9 | 515.5 | 613.2 | 753.7 |
| **3** | | | 0.2725 | | | 0.5709 | | | 0.9545 | | 1.596 | | | 3.343 | | 10.3 | | | 18.4 | | 27.55 | | | 41.25 | | 73.71 | | | 2.564 | | 7.586 | | 11.08 | 14.57 | 19.59 | 183.8 | 403 | 555.3 | 707.7 | 926.9 |
| **4** | | | 0.1991 | | | 0.3971 | | | 0.6416 | | 1.037 | | | 2.068 | | 11.89 | | | 17.02 | | 21.83 | | | 28 | | 40.06 | | | 2.694 | | 7.624 | | 11.05 | 14.48 | 19.41 | 374.3 | 467.3 | 532 | 596.7 | 689.7 |
| **5** | | | 0.2826 | | | 0.6071 | | | 1.033 | | 1.758 | | | 3.778 | | 7.571 | | | 17.38 | | 30.96 | | | 55.17 | | 126.6 | | | 2.956 | | 9.125 | | 13.41 | 17.7 | 23.87 | 221.8 | 405.3 | 532.9 | 660.4 | 843.9 |

***Table S3:*** Risk differences for pregnancy outcomes between subgroups of low (≤25th percentile) versus normal-high (>25th percentile) hormone levels on day of FET stratified by day of embryo transfer (days 2-3 versus 4-5) (abbr.: A, DYD = dydrogesterone; B, DHD = 20α-dihydrodydrogesterone; C, Progesterone = progesterone; D, E2 = estradiol).
